# Stressed Mothers Receiving Infant Mental Health-Based Early Head Start Increase in Mind-Mindedness

**DOI:** 10.3389/fpsyg.2022.897881

**Published:** 2022-06-01

**Authors:** Holly E. Brophy-Herb, Hailey Hyunjin Choi, Neda Senehi, Tiffany L. Martoccio, Erika London Bocknek, Michal Babinski, Stephen Krafchak, Courtney Accorsi, Roxanna Azmoudeh, Rachel Schiffman

**Affiliations:** ^1^Department of Human Development and Family Studies, Michigan State University, East Lansing, MI, United States; ^2^Department of Childhood Education and Family Studies, Missouri State University, Springfield, MO, United States; ^3^Department of Human Development and Quantitative Methodology, University of Maryland, Rockville, Rockville, MD, United States; ^4^Department of Educational Psychology, Wayne State University, Detroit, MI, United States; ^5^Department of Biology, Michigan State University, East Lansing, MI, United States; ^6^Department of Psychology, Michigan State University, East Lansing, MI, United States; ^7^Lyman Briggs College, Michigan State University, East Lansing, MI, United States; ^8^Department of Genomics and Molecular Biology, Michigan State University, East Lansing, MI, United States; ^9^College of Nursing, University of Wisconsin, Milwaukee, WI, United States

**Keywords:** Early Head Start, infant mental health, mind-mindedness, parenting stress, toddlers

## Abstract

Maternal mind-mindedness is a characteristic of supportive parenting and contributes to many positive social–emotional outcomes in early childhood. However, there is limited knowledge of mind-mindedness among parents experiencing parenting stress from low-income settings. This is a critical gap in evidence given the robust role of supportive parenting in children’s development and the capacity of home-based interventions to improve children’s outcomes through enhancing supportive parenting. This study examined: (1) maternal mind-mindedness, operationalized as mothers’ appropriate mind-related comments (MRC), across toddlerhood in mothers of toddlers who participated in infant mental health (IMH) based Early Head Start (EHS) services; and (2) whether parenting stress moderated EHS program effects on appropriate MRC over time. Data from a primarily White midwestern site in the United States were collected at study enrollment and when toddlers were 14-, 24-, and 36-months of age (*N* = 152; mothers *M*_*age*_ = 22.4 years, SD = 5.1; toddlers *M*_*age*_ = 14.4 months, SD = 1.3; 51% females). Data included parent-completed questionnaires and observed parent–child interactions, which were coded for MRC. Although there were no main effects of EHS programming on mothers’ appropriate MRC over time, multilevel growth curve modeling indicated that parenting stress moderated EHS effects on mothers’ appropriate MRC over time. Among mothers with greater parenting stress, those who received IMH-based EHS services demonstrated greater proportions of MRC over time as compared to mothers with greater stress in the control group. IMH-based parenting interventions that target parenting stress may promote appropriate MRC in low-income populations during toddlerhood.

## Introduction

Parental mind-mindedness is a form of parental mentalization characterized by parents’ proclivity to treat their children as psychological agents (Meins, 2013). Parents who mentalize about their children attend to their young children’s mental states (e.g., thoughts, emotions, intentions), interpret mental states as underlying explanations for children’s behaviors, and make mind-related comments (MRC) to children about their mental states (Meins et al., 2001). Interactional mind-mindedness, assessed during parent–child interactions, is thought to be particularly salient to infants and very young children under the supposition that infants (e.g., Meins et al., 2001, 2012) and toddlers (e.g., Colonnesi et al., 2019) do not yet have a full complement of verbal skills to communicate their mental states, leaving it necessary for parents to interpret children’s mental states. Mind-mindedness is a two-dimensional construct indicated by “appropriate” MRC (i.e., those that reflect parents’ accurate or attuned interpretations of children’s mental states) and “non-attuned” MRC (i.e., inaccurate interpretations) (Meins, 2013). Although parents may talk to their children during play, MRC are a unique form of speaking which reflects the parent’s awareness of the child’s mental states. This awareness may be articulated through the parent’s use of mind-related language to reference the child’s mental states.

Importantly, mind-mindedness is a cognitive-behavioral trait (Meins et al., 2011a; Kirk et al., 2015), characterizing a relational construct that is a quality of close relationships (Meins et al., 2014; Larkin et al., 2021) and distinct from general parenting sensitivity and supportiveness (e.g., see Meins et al., 2002, 2003, 2012, 2013). It is a particular form of mentalization that characterizes the parent’s psychological orientation to the child’s mental states underlying observable behaviors (Meins, 1997; Meins et al., 2014). In studies to date, most of which are limited to middle to upper income samples of parents with infants and toddlers, appropriate MRC comprise roughly 3–11% of parental verbalizations to young children during free-play (e.g., Colonnesi et al., 2019). Although MRC comprise a relatively small proportion of all parental talk with children in play, they make unique contributions to children’s developmental outcomes. For example, appropriate MRC (heretofore referred to as appropriate MRC) are associated with positive parent and child outcomes, including parental sensitivity, attachment security, children’s theory of mind, children’s language skills, and children’s executive functioning (see McMahon and Bernier, 2017 for a review and Aldrich et al., 2021 for a meta-analysis).

Non-attuned MRC account for approximately 1–3% of verbalizations (Meins et al., 2012, 2013; Kirk et al., 2015; Colonnesi et al., 2019; Giovanelli et al., 2020). A few studies have shown associations between non-attuned comments and infants’ less optimal physiological emotion regulation (Zeegers et al., 2018), more extreme negative emotional displays during still face paradigms (McMahon and Newey, 2018), and attachment insecurity (Meins et al., 2012). However, non-attuned comments are generally not associated with insensitivity (e.g., see Zeegers et al., 2019) or with children’s developmental outcomes (see meta-analysis by Aldrich et al., 2021), underscoring the orthogonal dimensionality of appropriate and non-attuned comments (Colonnesi et al., 2019).

The lack of mind-mindedness research among diverse samples, including parents experiencing poverty, limits the extent to which findings about mind-mindedness may or may not generalize across populations. Findings from a recent meta-analysis (Aldrich et al., 2021) suggest that mind-mindedness may differ by parental socioeconomic status (possibly lower mind-mindedness among parents in lower-income settings). Such suppositions underscore the need for research among lower-income populations who often experience greater contextual stressors. Importantly, limited research suggests that appropriate MRC may play a protective role in children at elevated risk for poor behavioral outcomes due to the chronic stressors of poverty (e.g., Meins et al., 2013, 2019). For example, Meins et al. (2013, 2019) found that appropriate MRC buffered the negative effects of poverty on preschoolers’ internalizing and externalizing behaviors and was associated with school-aged children’s reading and math achievement. Similarly, in prior work (Brophy-Herb et al., 2015) we found that mothers’ explanations of emotions during parent–child interactions were related to reductions in toddlers’ externalizing behaviors, but only for toddlers from households with greater demographic adversity. Additionally, in toddlers from families experiencing low income, maternal tendency to describe their toddlers with mental agency (termed representational mind-mindedness) predicted greater self-regulation 6 months later (Mckelvey et al., 2015), an established buffer against cumulative adversity (Bridgett et al., 2015; Fernandez et al., 2016). Pursuant to the benefits of appropriate MRC for parenting and young children’s developmental outcomes, specialized programs targeting parental mentalization have emerged in recent years (e.g., Potharst et al., 2017; Schacht et al., 2017; Riva Crugnola et al., 2021; Zeegers et al., 2019). However, despite theoretical relevance, little empirical evidence has documented how community-based parenting programs, such as Early Head Start (EHS), may be related to parents’ mind-mindedness, particularly among parents contending with the stressors of poverty.

### Promoting Supportive Parenting

Parenting education and support programs broadly seek to enhance parental functioning and supportive caregiving to promote children’s early development. One such program is EHS, a federally funded two-generational program in the United States serving parents and children birth to age three, generally in populations experiencing poverty. Interestingly, some EHS programs employ attachment-based parenting education that is similar to targeted interventions that focus on improvements in parental mentalization, including mind-mindedness. In particular, infant mental health (IMH) based EHS home visiting models (used in the current study) focus on concepts similar to those included in mind-mindedness interventions, including a strong focus on building parental awareness of very young children’s mental states. However, EHS evaluations have more generally focused on program impacts on broad parenting constructs, such as parental supportiveness in play (e.g., Love et al., 2002). Evaluations to date have not examined program effects on mind-mindedness. This gap in knowledge is not necessarily surprising given improvements in mindedness have been tied almost exclusively to targeted interventions that specifically focus on parental mentalization. It is worth noting, however, that such interventions share similarities with IMH-based EHS home visiting models in that they focus on improvements in parents’ awareness of children’s mental states. To illustrate this point, we begin with a description of interventions targeting improvements in mind-mindedness and then draw parallels to IMH-based intervention programs.

Zeegers et al. (2019) used a quasi-experimental design to test associations between participation in the Mindful with Your Baby/Toddler program (Potharst et al., 2017) and mothers’ post program parenting stress and mind-mindedness, assessed during parent–child interactions. The program specifically focused on mothers with mental health concerns (e.g., mood disorders, anxiety, post-traumatic stress disorder) and used mindfulness techniques to enhance mothers’ attention to their own and their infants’ and toddlers’ mental states. At post intervention, mothers made fewer non-attuned MRC, although there were no significant effects on appropriate MRC.

Schacht et al. (2017) piloted a single session video feedback intervention aimed at promoting mothers’ mind-mindedness in mothers with mental health disorders. During feedback sessions, interventionists discussed observed changes in infants’ mental states as underlying behaviors, pointed out opportunities to comment on infants’ mental states, and encouraged mothers to reflect on their infants’ mental states. Mothers who received the intervention displayed significantly fewer non-attuned comments to infants during parent–child interactions and there were trend level increases in appropriate MRC.

Riva Crugnola et al. (2021) tested the effectiveness of the Promoting Responsiveness Emotion Regulation and Attachment in Young Mothers and Infants, an attachment-based parenting intervention using video feedback, on adolescent mothers’ mind-mindedness. As compared to the mothers who did not receive the intervention, mothers in the intervention group demonstrated more appropriate MRC, and fewer non-attuned MRC, at the post assessment. In one of the few other mind-mindedness interventions among community samples, Larkin et al. (2019) paired a psychoeducation session with the BabyMind app, a smartphone application designed to promote mind-mindedness. The app included a variety of features focused on building mind-mindedness including depictions of infants’ mental states, journal prompts to elicit mothers’ curiosity about their infants’ mental states, and developmental information for mothers. All messages and prompts were personalized to each parent–infant dyad (e.g., the child’s name, specific age, etc.). Mothers began the intervention during pregnancy and were observed during parent–child interactions when their infants were 6 months old. Mothers in the intervention group demonstrated greater appropriate MRC as compared to mothers in a control group, and the app was effective with younger and older mothers.

Like IMH-based EHS home visiting models, a common element in each of these interventions was a focus on drawing parents’ attention to their children’s mental states. Notably, most of the mind-mindedness interventions described primarily involved clinical samples of parents or adolescent parents, leaving questions as to whether community samples of parents might also benefit from interventions to enhance mind-mindedness. Interestingly, several attachment-related interventions aimed at enhancing parental mentalization largely grew out of IMH-based home visiting programs. For example, Slade et al.’s (2005, 2020) Minding the Baby intervention was designed to promote secure parent–infant relationships through increasing mothers’ capacities to identify and accurately interpret their own and their infants’ mental states and to consider the ways in which mental states underlie behavior. First used with mothers with a history of childhood trauma living in low-income settings, the program aimed to support mothers’ curiosity about and interpretation of the baby’s thoughts, feelings, and needs. Through a series of studies, the program has shown positive effects in increasing mothers’ mentalization about their infants and promoting attachment security (e.g., Slade et al., 2020). Fonagy et al. (2002) noted that the capacity to understand mental states in oneself and in the baby grows out of our early interpersonal experiences, namely the early relationship experiences “particularly the experience of being known and understood by one’s caregivers” (Slade et al., 2005; p. 76). Fraiberg et al. (1981, 2003), largely credited with founding the field of IMH, posited that disruptions in early relationships hamper parents’ capacities to imagine their infants’ experiences and contribute to parents’ misunderstandings, distortions, and misattributions of infants’ behaviors and the underlying mental states. Parent–infant psychotherapy was developed as a treatment model to address disruptions in the parent–infant relationship by supporting the parent’s capacity to hold representations of the baby that are coherent, organized, and reflect accurate interpretations of the baby’s mental states. Techniques often involve carefully observing the ways and times in which pregnancy, birth, and early parenting experiences evoke the parent’s prior trauma and/or painful relationship history (Weatherston et al., 2020). Recent research has demonstrated that IMH-based visiting models are associated with mothers’ greater sensitivity in interactions with their infants and toddlers (Rosenblum et al., 2020) and with mothers’ increased mentalization, to date, assessed as parental reflective functioning (Stacks et al., 2019, 2022); reflective functioning refers to the parent’s capacity to hold awareness of the infant’s internal mental states (Slade, 2005). Hence, a next step in this evolving research is to examine any potential IMH-based intervention effects on mothers’ mentalization, assessed as their appropriate MRC in interactions with their children.

### Infant Mental Health-Based Early Head Start Programs

The current study utilized data used in an IMH-based home visiting EHS program. As noted above, the IMH model has been integrated into some home-based EHS programs (Mckelvey et al., 2015). Home visiting programs that employ an IMH-based frame typically included home visitors who are clinicians trained in social work or a related field and trained in IMH; IMH home visitors also typically receive regular reflective supervision (Weatherston et al., 2020). Like the intervention programs designed to promote parental mind-mindedness, IMH-based home visiting practices are characterized by the clinician’s intentional observations of the parent and baby, including interpretations of their mental states, such as infants’ emotional needs and intentions. Such observations carefully guide the clinician in working with the parent and baby, which includes supporting parents’ curiosity about and observations of their infants’ mental states (see Weatherston et al., 2020). As Weatherston explained “By observing and listening, staying open to both the pleasures and the pains, the therapist joins the infant and parent in creating a space where they come to know one another. It is in this safe place that the IMH-based home visitor therapist and parent together can observe, wonder about, explore, and understand the attachment relationship between the parent and infants” (p. 168). Such work, by nature, involves reflecting together on the infant’s and the parent’s mental states. Moreover, clinicians typically observe and comment on parent’s mental states in their interactions with the parent. Such a parallel process mirrors in the clinician–parent relationship the hopes for the parent–baby relationship (Weatherston and Osofsky, 2009). In addition to attending to material needs, providing emotional support, offering developmental guidance, and case management (all core components of the IMH-based model), the process of building an alliance with the parent and the use of parent–infant psychotherapy (additional core components of IMH-based home visiting) may both support and model parental mind-mindedness.

Given the shared theoretical roots, we posited that IMH-based EHS home visiting may promote appropriate MRC in ways similar to mind-mindedness interventions. Results from such work would have important implications for optimizing EHS programs in support of parenting. Moreover, it may be that mothers experiencing greater stress in their parenting roles particularly benefit from interventions aimed at enhancing their awareness and understanding of their young children’s internal experiences. This supposition is aligned with intervention literature suggesting that higher risk parents may benefit more robustly from interventions. For example, Gardner et al. (2010) reported that parenting intervention program effects on child behaviors were more robust in the context of greater parental depressive symptoms. Other parenting interventions, however, found that parenting stress and depression did not moderate parenting intervention effects on parenting behaviors (e.g., Theise et al., 2014). Such mixed results leave the question of moderation open to investigation and led us to examine parental distress in the current study.

### Current Study

The current study pursued two aims. The first aim was to examine the effects of enrollment in IMH-based EHS intervention on mothers’ appropriate MRC observed during parent–toddler interactions across toddlerhood when children were 14, 24, and 36 months of age for participants in the National Early Head Start Research and Evaluation Study (Love et al., 2005). We posited that mothers enrolled in IMH-based EHS, would demonstrate increased appropriate MRC over time relative to mothers who did not receive IMH-based EHS services. A second aim tested whether any EHS effects on appropriate MRC over time were more robust for mothers who reported greater parenting stress. We expected that mothers with greater parenting stress who received the intervention aimed at enhancing their understanding of their children would show increased appropriate MRC as compared to mothers with stress in the control group.

Consistent with the existing literature on mind-mindedness, we controlled for toddler temperament (e.g., Demers et al., 2010; Meins et al., 2011b; McMahon and Newey, 2018), and child age and sex (e.g., McMahon and Newey, 2018). We also covaried child language skills as language competencies could theoretically contribute to or elicit mothers’ MRC. Similarly, we controlled for maternal age. We did not pose specific hypotheses relative to child effects on MRC nor did we pose questions about maternal demographic characteristics relative to MRC. Although associations between mind-mindedness and these covariates warrant further examination, such questions were not central to the primary purpose of the study.

## Materials and Methods

### Participants

Data for these analyses were drawn from the Early Head Start Research and Evaluation Project (EHSREP), a United States nationally representative randomized controlled trial (RCT) with 3,001 participants in which low-income families randomized to the EHS intervention group or to a usual care control group (see Love et al., 2005). The EHSREP was comprised of multiple research sites around the country. The current study utilized data from 152 parent–toddler dyads from a midwestern research site. At this research site, videotaped parent–child interaction data were coded for parental mind-mindedness; 52% (*n* = 79) of participants at this site were randomly assigned to receive EHS services and 48% (*n* = 73) were randomized to a control group. The midwestern site used in the current study administered weekly EHS home visits and employed an IMH-based home visiting model. Although some EHS sites in the national EHSREP were center-based programs providing early childcare and education, the current study’s site was solely home-based and did not include childcare experiences. At random assignment, mother’s average age was 22.4 years, SD = 5.1, range 15–38 years. Maternal race was self-reported as primarily 69% White (*n* = 105) or 15% Black (*n* = 23). At enrollment, toddlers’ mean age was 14.4 months, SD = 1.3, range 12.61–20.20 months; 51% of toddlers were females and most (61%, *n* = 93) were first born children. Most mothers reported holding less than a high school diploma (35%) or the equivalency to a high school diploma (31%) while 22% had some college education. Annual family income averaged $9,436 (median = $7,714) and ranged from $0 to $52,000.

Written informed consent was obtained prior to data collection. Study protocols and activities were approved by the Human Research Protection Program at (Mckelvey et al., 2015) University and were compliant with the ethical standards of the American Psychological Association and the World Medical Association (Declaration of Helsinki).

### Procedure

Data were collected in the home by trained research assistants and included videotaped parent–child interactions during semi-structured play and administration of parental questionnaires. Observations of semi-structured play were video recorded during home visits when toddlers were 14, 24, and 36 months old as part of the EHSREP protocol and were later coded for mind-mindedness.

### Measures

#### Maternal Mind-Mindedness

Maternal mind-mindedness was coded from the Three-Bag-Assessment (Ware et al., 1998), a semi-structured play task. During the task, mothers were given three bags of toys and asked to play with their toddlers for 10 min, beginning with the first bag. Bags at each time point included developmentally appropriate toys including board books, pretend play materials such as kitchen set props, and puzzles. For a full description, see Kopack Klein et al. (2016).

To assess mind-mindedness, videotaped interactions at each time point were transcribed verbatim and coded per procedures from Meins and Fernyhough (2015) using the Mind-Minded Coding Manual, Version 2.2. MRC were identified using the transcribed play episodes and included references to toddlers’ cognitions (e.g., thoughts, knowledge; “You know how to get the toy lid back on”) and mental processes (e.g., recognition, remembering, decision making; “You decided to play with the pots and pans first”), emotions (“You’re happy today”), preferences and desires (“You like to play with the cooking set”), and intentional acts on people’s beliefs (e.g., joking, teasing; “You’re teasing me”). “Speaking” for the toddler, in which the mother verbalized what she imagined the toddler might be thinking or feeling, was also coded (e.g., “Play with me, mama”).

Next, MRC were coded from the videotaped interactions as appropriate or non-attuned by research assistants who were blind to study conditions and to all other measures. Using criteria established by Meins and Fernyhough (2015), MRC were coded as appropriate if: (a) the coder evaluated the mother’s interpretation of the toddler’s mental state to be accurate; (b) the comment linked the toddler’s current activity with related past or future events (e.g., such as referring to the toddler’s prior knowledge or preference for a particular toy); (c) the comment was a suggestion for a new play activity after a lull in the interaction (e.g., “Would you like us to read the book next?”). To account for maternal verbosity, a proportion score, reflecting the proportion of appropriate MRC out of the total number of all comments made by the mother to the toddler during the play interaction, was used in the current analyses.

Coders were trained by the first and second author until they attained reliability set at intra-class correlation (ICC) of 0.75 or greater. A random sample of 20% of videotaped were double coded with intercoder reliability in the same range. Specifically, ICCs for total MRC ranged from 0.93 to 1.00 across time points; from 0.92 to 0.97 for appropriate MRC over time, and from 0.90 to 0.91 for non-attuned MRC over time.

#### Parenting Stress

Perceived stress in the parenting role was assessed with the distress subscale from the Parenting Stress Index—Short Form (PSI/SF; Abidin, 1995) collected from mothers at the 14-month assessment. Twelve items measuring stress in the parenting role were rated on a 5-point scale from (1) strongly agree to (5) strongly disagree with higher scores reflecting greater stress (*a* = 0.79 in the current study). Example items of stress subscale included “Being a parent is manageable, and any problems are easily solved” or “I feel trapped by my responsibilities as a parent” Scores ranged from 12 to 57 (*M* = 30.23, SD = 9.52). Twenty-two percent of mothers reported parenting stress in the cutoff range (>36) described by Abidin (1995), indicating high stress.

#### Covariates

Maternal age at study enrollment, toddler’s age, sex, temperament, and productive vocabulary were used as covariates. Temperament was measured by the emotionality subscale from the Emotionality, Activity, Sociability, and Impulsivity (EASI) Temperament Survey (Buss and Plomin, 1984) at 14 months. Mothers rated their children on emotionality *via* five items with responses ranging from (1) not very typical of the child to (5) very typical of the child (*M* = 3.00, SD = 0.90, range = 1.20–5.00; *a* = 0.74). Children’s vocabulary was assessed *via* the parent-reported MacArthur Communicative Development Inventories (Fenson et al., 2000; Jahn-Samilo et al., 2001) when children were 14- and 24-months old. At 14 and 24 months, parents reported on their children’s vocabulary production by indicating how many of 100 listed words their children could understand and could say. Per the instrument manual, the productive vocabulary score is normed for child age and ranges from 0 to 100 (Fenson et al., 1994).

### Analytic Plan

Preliminary analyses used *t*-tests and Chi-square analyses to test for any differences in baseline demographic characteristics between EHS and control groups. We used multilevel modeling for the principal analyses to examine individual growth trajectories of maternal appropriate MRC with their toddler across the three time points. For the time index variable, the 14-month (baseline) time point was coded as 0, with the following time points coded as 1 and 2 (24 and 36 months, respectively). To address the study research questions, we first ran an unconditional growth model (aim 1). We then used higher-order interaction between Level 2 time-invariant variables to examine whether parenting stress moderated any RCT group effects on appropriate MRC. Where appropriate, simple slopes were calculated as equal to the slope of the predictor variable when the RCT grouping variable was coded as −1 and 1 for the comparison and EHS group (aim 2), respectively. The moderating variable was probed at low and high levels of parenting stress using the 40th and 80th percentiles, as probing at low and high levels of the moderator is recommended as a best practice approach (Hayes and Little, 2018). The method of probing along the percentiles of the moderating variable confirmed that the probed points were within the range of the observed data (Hayes and Little, 2018). Independent variables were standardized, and the higher-order interaction term was created by multiplying the Level 2 variables in the term. Models were conducted in M*plus* v.8 (Muthén and Muthén, 1998–2017), using maximum likelihood with robust standard errors (MLR) estimation. Missing data were handled using full-information maximum likelihood (FIML). Regarding covariates, maternal age was positively related to the intercept for appropriate MRC (*B* = 0.007, SE = 0.003, *p* = 0.024) but did not interact with group status and was not included in the final model. The addition of maternal age effect on variation in linear slopes did not alter the higher-level interaction between RCT group and parenting stress. Toddler vocabulary skills, temperament, age, and toddler sex were not significant in model analyses. Vocabulary and temperament were subsequently dropped from the final models in favor of model parsimony. Although not significant, toddler age and sex were retained as they are standard, empirically informed covariates in many studies of parent–child interactions.

Although our interest was in appropriate MRC, we computed models for non-attuned MRC. Non-attuned MRC accounted for just 2% of all maternal comments during play, similar to values reported in the literature (e.g., Zeegers et al., 2019; Suttora et al., 2021). Models for non-attuned MRC were not significant. Therefore, we do not provide additional information on non-attuned MRC, although they are available upon request.

## Results

Table 1 presents descriptive demographics of participants and study measures at baseline, Table 2 includes correlations among study variables. There were no significant differences in baseline characteristics between mothers in the EHS or control groups. At the baseline assessment across the sample, approximately 5% of all maternal comments made during the play interactions were MRC, of which 3–5% were appropriate MRC (see Table 1). Although not a focus of the current study, the means and standard deviations of MRC by ethnic-racial groups are available in Appendix A; there were not significant differences.

**TABLE 1:** Frequencies, means, and standard deviations for study variables.

	Whole sample		EHS group		Control group	
Characteristic	*n* (%)	M (SD)	*n* (%)	*M* (SD)	*n* (%)	*M* (SD)
Toddler age (in months) at the 14 month assessment		14.41 (1.28)		14.99 (1.20)		14.80 (1.28)
**Toddler sex**						
Females	78 (51%)		41 (52%)		37 (51%)	
Males	74 (49%)		38 (48%)		36 (49%)	
Toddler negative emotionality		3.00 (0.90)		2.97 (0.93)		3.02 (0.87)
Toddler productive vocabulary, 14 months		12.51 (11.22)		13.58 (11.97)		11.30 (10.27)
Toddler productive vocabulary, 24 months		53.04 (25.94)		57.30^+^ (24.98)		48.09 (26.35)
Maternal age (in years) at study enrollment		22.40 (5.07)		22.23 (5.04)		22.59 (5.13)
**Maternal race and ethnicity**						
White	105 (69%)		51 (65%)		54 (74%)	
Black	23 (15%)		13 (17%)		10 (14%)	
Latina	3 (2%)		1 (1%)		2 (3%)	
Other	6 (4%)		3 (4%)		3 (4%)	
Missing data	15 (10%)		11 (13%)		4 (5%)	
**Maternal education**						
< High school diploma	54 (35%)		27 (34%)		27 (37%)	
High school diploma	47 (31%)		26 (33%)		21 (29%)	
> High school diploma	33 (22%)		15 (19%)		18 (24%)	
Missing data	18 (12%)		11 (14%)		7 (10%)	
Annual family income in USD at study enrollment		9436.39 (7354.95)		8193.34 (6090.83)		10491.09^++^ (8176.13)
Parenting stress, clinical cutoff (>36)-14 months assessment	33 (22%)		18 (23%)		15 (21%)	
Parenting stress 14 months assessment		30.23 (9.52)		30.16 (10.04)		30.31 (8.99)
Proportion of appropriate MRC of all maternal comments made, 14 months		0.03 (0.03)		0.03 (0.03)		0.04 (0.04)
Proportion of appropriate MRC of all maternal comments made, 24 months		0.03 (0.03)		0.03 (0.03)		0.03 (0.03)
Proportion of MRC comments of all maternal comments made, 36 months		0.04 (0.02)		0.04 (0.02)		0.04 (0.02)
Total number of maternal comments at 14 months		96.38 (50.14)		103.39^+++^ (51.35)		88.78 (48.08)
Total number of maternal comments at 24 months		132.21 (50.83)		135.97 (50.58)		128.03 (51.18)
Total number of maternal comments at 36 months		128.90 (51.68)		130.41 (51.24)		127.02 (52.70)

*^+^p = 0.07; ^++^p = 0.09; ^+++^p = 0.11. There were no significant differences between EHS and control groups in demographic characteristics, parenting stress, child negative emotionality, or maternal mind-mindedness. Toddlers’ productive vocabulary was marginally (p = 0.07) greater for toddlers in the EHS group. Income was marginally (p = 0.09) higher for parents in the control group. The total number of parental comments at 14 months was marginally higher for parents in the EHS group (^++^p = 0.11).*

**TABLE 2:** Correlations among study variables.

Variables	1	2	3	4	5	6	7	8	9	10
1. Toddler age	−									
2. Toddler sex	0.11	−								
3. Toddler temperament	–0.08	–0.07	−							
4. Toddler productive language, 14 months	0.17*	0.07	–0.12	−						
5. Toddler productive language, 24 months	–0.04	0.12	–0.13	0.53***	−					
6. Maternal age	0.13	0.05	0.08	–0.06	–0.04	−				
7. Parenting stress, 14 months	–0.09	0.05	0.27***	–0.15	−0.15^†^	–0.00	−			
8. Maternal appropriate mind-related comments, 14 months	0.04	–0.08	–0.17	0.21*	0.31**	0.22*	–0.12	−		
9. Maternal appropriate mind-related comments, 24 months	0.00	0.02	–0.14	0.19*	0.11	0.16	–0.05	0.37***	−	
10. Maternal appropriate mind-related comments, 36 months	0.06	0.10	−0.18^†^	0.11	0.09	0.24*	–0.05	0.25*	0.36***	−

^†^p < 0.10; *p < 0.05; **p < 0.01; ***p < 0.001.

The unconditional model indicated an average initial MRC level of 0.032 (*B* = 0.032, SE = 0.003, *p* < 0.001). Effects for random intercepts (*B* = 0.001, SE = 0.000, *p* = 0.02) indicated variation for the level in appropriate MRC across mothers. We thus included terms for random intercepts and random linear slopes in the subsequent conditional model.

Table 3 includes parameter estimates for the conditional model that examined main and moderation effects on variation in the intercept and linear slope for appropriate MRC. Moderation results indicated that RCT group membership and parenting stress interacted to predict marginally significant variation in the intercept for appropriate MRC (*B* = −0.005, SE = 0.003, *p* = 0.088). Furthermore, the interaction of RCT group and parenting stress predicted the variation in linear slopes of MRC (*B* = 0.005, SE = 0.002, *p* = 0.004). The effect for RCT group, independently, however, was not significant.

**TABLE 3:** Parameter estimates for multilevel models predicting maternal appropriate mind-related comments.

	Unconditional model				Conditional model			
Parameter	Estimate	SE	*z*	*p*	Estimate	SE	*z*	*p*
**Fixed effects**								
Intercept	0.032	0.003	11.484	< 0.001	0.033	0.003	11.420	< 0.001
Time	0.003	0.002	1.421	0.155	0.003	0.002	1.624	0.104
**Effects on intercept**								
Toddler age					0.003	0.002	1.532	0.125
Toddler sex (females)					0.001	0.002	0.600	0.548
Maternal age					0.007	0.003	2.407	0.016
RCT group (EHS)					0.000	0.003	–0.146	0.884
Parenting stress (PS)					0.001	0.003	0.330	0.741
EHS × PS					–0.005	0.003	–1.707	0.088
**Effects on linear slope**								
Maternal age					–0.003	0.002	–1.605	0.109
RCT group (EHS)					0.000	0.002	0.026	0.979
Parenting stress (PS)					–0.002	0.002	–0.990	0.322
EHS × PS					0.005	0.002	2.886	0.004
**Random effects**								
Level 2 (between-person)								
Intercept	0.001	0.000	2.327	0.020	0.001	0.000	2.190	0.028
Linear slope	0.000	0.000	0.993	0.321	0.000	0.000	1.072	0.284
Intercept with linear slope	0.000	0.000	–1.718	0.086	0.000	0.000	–1.644	0.100
Level 1 (within-person)								
Residual	0.001	0.000	4.212	< 0.001	0.001	0.000	4.450	< 0.001

*Unstandardized estimates are presented.*

**FIGURE 1:**
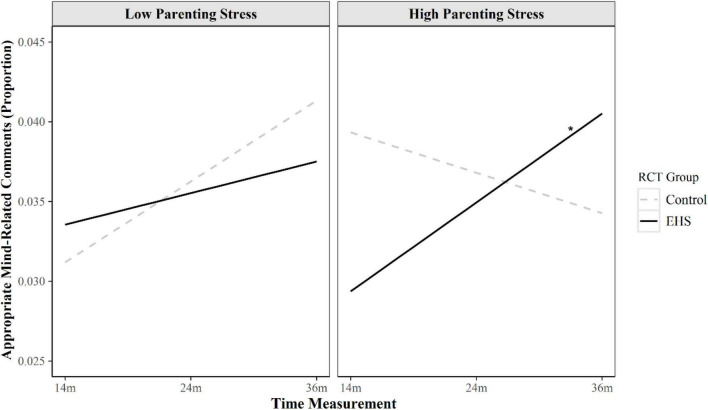
Conditional growth curves of appropriate MRC by RCT group and parenting stress. This figure illustrates trajectories of appropriate MRC over time for mothers in the EHS intervention or control group with low (40th percentile) and high (80th percentile) parenting stress. Among mothers experiencing high parenting stress, those who received infant mental health informed EHS displayed increases in appropriated MRC over time relative to control group. However, among mothers experiencing low parenting stress, there was no significant difference in appropriate MRC growth between EHS and control mothers. *p < 0.05.

Given the current study’s aim on longitudinal variation in appropriate MRC, simple slopes were calculated for the linear slopes only. As shown in Figure 1 and as indicated by tests of simple slopes, for mothers in both the EHS (*B* = 0.002, SE = 0.002, *p* = 0.376) and control (*B* = 0.005, SE = 0.003, *p* = 0.117) groups, low parenting stress indicated no significant variation in MRC over time, regardless of RCT group status. Among control mothers with high parenting stress, the variation in linear slope of appropriate MRC was also not significant (*B* = −0.002, SE = 0.004, *p* = 0.508). Notably, however, this effect was significant among EHS mothers with high parenting stress (*B* = 0.006, SE = 0.002, *z*-value estimate = 2.89, *p* = 0.019). The finding indicates that among mothers experiencing high parenting stress, those who received IMH based EHS services displayed increases in proportion of appropriated MRC over time relative to control group.

## Discussion

Although there were no main effects of IMH-based EHS programming on appropriate MRC over time, parenting stress moderated IMH-based EHS intervention effects. Specifically, among mothers with greater parenting stress, those who received IMH-based EHS programming demonstrated significantly greater proportions of appropriate MRC over time than did mothers with greater parenting stress in the control condition.

The more robust effects for highly stressed mothers in EHS (relative to the control group) are in line with the literature underscoring links between parenting stress and EHS participation and between parenting stress and mind-mindedness. Parenting intervention programs have shown success in reducing parenting stress and helping parents to cope with parenting stress. For example, other studies utilizing data from the Early Head Start Research and Evaluation Study have demonstrated that parents in EHS are less likely to report chronically high parenting stressthan their peers not receiving EHS services (Chang and Fine, 2007) and shown the buffering effects of EHS on links between parenting stress and child outcomes (e.g., Ayoub et al., 2011). Mothers in EHS in the current study may have received more support to manage and reduce their parenting stress than did mothers in the control group. As noted previously, greater parenting stress is related to reduced optimal parenting; hence it is possible that mothers who learned how to cope with parenting stress were better able to engage in greater mind-mindedness with their toddlers. Although beyond the scope of the current study, this supposition is also in line with research underscoring the negative associations between higher parenting stress and lower quantity (McMahon and Meins, 2012) and quality (Demers et al., 2010) of mind-mindedness. With support in managing stress, mothers may have been better able to attend of the mentalization technique typically modeled by IMH-based home visiting. Home visitors who use IMH practices tend to engage in mind-mindedness themselves as they notice, interpret, and comment on mothers’ and toddlers’ mental states while working with mothers and toddlers.

Likewise, the use of parent–infant psychotherapy practices invites mothers to notice and wonder about their toddlers’ mental states (Weatherston et al., 2020). Given that case notes detailing the use of IMH-based principles and strategies in home visits were not available, we cannot know for certain whether or how IMH-based practices contributed to increased mind-mindedness in mothers with greater stress. However, our suppositions are theoretically plausible, and further research is warranted to investigate these associations. We also acknowledge that declines in stress may have contributed to increase mind-mindedness aside from EHS programming. Although it was not the purpose of the current study, future work might also focus on potential bidirectional associations between mind-mindedness and stress over time. We acknowledge that greater mind-mindedness may contribute to less parenting stress under the supposition that better understanding of children’s mental states may make parenting less stressful. For example, McMahon and Meins (2012) found that stress mediated the effects of mind-mindedness on other observed parenting behaviors. It is also plausible that a bidirectional relationship exists such that that mind-mindedness lessens stress and less stress promotes supportive parenting practices including mind-mindedness with reciprocal relations continuing over time.

Our moderation finding is in line with other IMH-based intervention studies that have reported moderated effects rather than main intervention effects. For example, Stacks et al. (2022) reported increased mentalization capacity (reflective functioning) in mothers who were experiencing low-income and participating in IMH-based home visiting, specifically for mothers who received a greater dosage of IMH-based home visits from highly experienced clinicians. These findings point to the potentially unique contexts in which program effects may be more evident or more robust.

Still the lack of EHS main effects on mind-mindedness was somewhat surprising given the IMH-based focus of this EHS program. To date, IMH-based home visiting has been linked to increases in parental sensitivity (Rosenblum et al., 2020) and reflective functioning (Stacks et al., 2022). However, the techniques used in IMH-based home visiting, although based in mentalization, may not be transparent enough to parents to intentionally facilitate parental mind-mindedness. Intervention programs that have reported effects on mind-mindedness use specific strategies and discussion with parents centered specifically on the concept of mind-mindedness. For instance, interventionists in Schacht et al.’s (2017) study watched videotaped parent–infant interactions with mothers and focused discussion specifically on infants’ mental states. It may be that transparent strategies directly focused on mind-mindedness are more effective in promoting mothers’ mind-mindedness.

Another explanation for the lack of EHS main effects on mind-mindedness may be that, unlike targeted mind-mindedness interventions, home visiting services include a great deal of additional content in home visits. Home visitors also engage in case management, general parenting education, developmental guidance relative to normative child developmental milestones, and child health, among other things. The sheer content of information addressed in home visits may limit the time home visitors have to focus on aspects of parental mentalization.

Finally, we turn to some discussion of associations between covaried maternal and child characteristics and MRC. Greater maternal age was positively related to MRC at the 14- and 36-months assessment point. Mothers in the current study varied widely in age with both adolescent mothers and adult mothers represented in the sample. Although adolescent mothers are a heterogenous group, research tends to suggest that early parenting poses risks to parenting quality (Lee, 2009). Similarly, Riva Crugnola et al. (2018) found that adolescent mothers made fewer appropriate MRC to their infants as compared to older mothers. Hence, the associations we report in the current study are aligned with the existing literature.

Maternal stress and child temperament were associated, a finding that replicates a vast body of prior work (e.g., Ortiz and Barnes, 2018). It is also important to note that most of the few existing intervention programs to enhance mind-mindedness have focused on mothers with serious mental health concerns (Potharst et al., 2017; Schacht et al., 2017) and adolescent parents (Riva Crugnola et al., 2021). Although beyond the scope of current findings, further investigations on these associations are warranted as children’s difficult temperament in interaction with other maternal stressors may have significant implications understanding intervention effects and planning subsequent program supports for mothers and toddlers.

Interestingly, toddlers’ language skills and MRC were correlated when children were 14 and 24 months old, suggesting the possibility of directional or perhaps bidirectional associations. It is possible that exposure to greater mental state vocabulary promotes young toddlers’ vocabulary, while it may also be that toddlers’ language skills elicit MRC. Notably, by the time toddlers were 36 months old, associations between age and MRC were no longer evident, suggesting that young toddlerhood may be a more developmentally salient time to examine directional and bidirectional associations. A recent study showed no associations between mind-mindedness in infancy and language skills in infancy or toddlerhood among a sample of Swedish infants and parents (Nyberg et al., 2021) while an earlier study showed positive associations between maternal mind-mindedness at 12 months and toddlers’ expressive language a year later (Laranjo and Bernier, 2013); it could be that mind-mindedness in early toddlerhood, rather than in infancy, plays a role in toddlers’ language, although this supposition is beyond the scope of this study. Such investigations reflect an interesting next step in the mind-mindedness literature, particularly given the emerging studies examining mind-mindedness and children’s language and cognitive outcomes in early childhood (e.g., Bernier et al., 2017; Aldrich et al., 2021).

### Limitations

Study limitations largely reflect lack of generalizability beyond the current study sample. First, EHS effects in the current study reflected the integration of the IMH model in EHS and cannot be generalized to all EHS programs. As noted previously, although we detected program effects for highly stressed mothers, we are unable to identify what specific aspects of the IMH-based home visiting EHS services contributed to these effects.

Second, we acknowledge the limitations on generalizability in this sample of mostly White mothers. Small sample sizes precluded the possibility of examining the validity of mind-mindedness among parents of color in a meaningful way. Further, in reviewing the literature, we found no studies of mind-mindedness specifically among diverse populations. The one study we found from Bernier and Dozier (2003) was a primarily (63%) Black sample of foster mothers (63%) and Black (67%) foster children, although race was not a significant element in the research questions posed which focused on associations between mind-mindedness and attachment security. Most studies that included Black parents or Latina parents were still primarily White samples and reflected mostly clinical samples of parents with mental health disorders or the parents of atypically developing children (among examples of recent studies are Pawlby et al., 2010; Kirk and Sharma, 2017; Schacht et al., 2017; Bigelow et al., 2018). While these studies made valuable contributions to the establishment of a literature on mind-mindedness, much remains to be understood about the mentalization experiences of parents of from diverse ethnic-racial groups (Fishburn et al., 2022). We acknowledge the ways in which the current study, similar to current literature of mind-mindedness, is conceptually and methodologically limited to majority White families. Our future investigative efforts will include research on ethnic-racial minority parents that aim to promote equity in research, including seeking to understand how constructs such as mind-mindedness may be conceptualized and/or function differently among parents from varying ethnic-racial groups. Finally, only a handful of studies have examined paternal mind-mindedness (e.g., Lundy, 2013; Gagné et al., 2018; Colonnesi et al., 2019; Miller et al., 2019; Planalp et al., 2019), highlighting the need to include fathers in mind-mindedness studies. Additionally, the current investigation is not well powered and limited in available data to examine potential mediational mechanisms that may underlie changes in maternal appropriate MRC over time. Thus, future studies in families facing adversity may examine how changes in parenting stress as well as changes in adversity-related stress may contribute to increases in appropriate MRC over time.

## Conclusion

To our knowledge, very few studies have examined observed mind-mindedness longitudinally, and we found no studies that tracked interactional mind-mindedness across the first 3 years. This longitudinal study of maternal mind-mindedness in a non-clinical, sample of mothers experiencing cumulative contextual adversity contributes to the growing literature on interactional mind-mindedness beyond infancy. Findings also shed light on the contexts (i.e., greater parenting stress) in which IMH parenting support programs may have a positive impact on maternal mind-mindedness in non-clinical samples.

## Author’s Note

Data for this study were obtained from the Early Head Start Research and Evaluation Project, which was supported by grant #90YF0010, Pathways Project: Research into Directions for Family Health and Service Use, from the Administration on Children, Youth, and Families, Department of Health and Human Services, RS, Ph.D., R.N., Principal Investigator, Michigan State University. The cross-site national evaluation data were collected under contract to Mathematica Policy Research, Inc., Princeton, NJ, United States, which was responsible for the national Early Head Start program evaluation under contract 105-95-1936 with the Administration for Children and Families, United States Department of Health and Human Services. Key MPR staff were John M. Love (Project Director), Ellen Kisker (Principal Investigator), and Jeanne Brooks-Gunn (Principal Investigator), under the supervision of the project manager for ACYF, Helen H. Raikes.

## Data Availability Statement

The maternal mind-mindedness datasets presented in this article are not readily available because they are subject to ongoing research. Other study data are publicly available from https://www.icpsr.umich.edu/web/pages/ICPSR/index.html. Requests to access this dataset should be directed to https://www.icpsr.umich.edu/web/pages/ICPSR/index.html. Requests to access the mind-mindedness data should be directed to the corresponding author.

## Ethics Statement

The studies involving human participants were reviewed and approved by Human Research Protection Program, Michigan State University. The patients/participants provided their written informed consent to participate in this study.

## Author Contributions

HB-H, HC, NS, and TM contributed to the conception and design of the study. TM conducted the data analyses. MB, SK, CA, and RA coded the data and reviewed the manuscript. HB-H, TM, NS, and EB wrote sections of the manuscript. RS reviewed and edited the manuscript and was a principal investigator of the Early Head Start Research and Evaluation Study in which the original data were collected. HB-H and HC trained data coders. HC read and edited the manuscript. All authors contributed to the article and approved the submitted version.

## Conflict of Interest

The authors declare that the research was conducted in the absence of any commercial or financial relationships that could be construed as a potential conflict of interest.

## Publisher’s Note

All claims expressed in this article are solely those of the authors and do not necessarily represent those of their affiliated organizations, or those of the publisher, the editors and the reviewers. Any product that may be evaluated in this article, or claim that may be made by its manufacturer, is not guaranteed or endorsed by the publisher.

## Appendix

**TABLE A1:** Appropriate MRC by Ethnic-Racial Group.

Mind-mindedness	*M* (SD)
**Proportion of appropriate MRC of all maternal comments made, 14 months**	
White (*n* = 85)	0.04 (0.03)
Black (*n* = 19)	0.04 (0.04)
Latina (*n* = 1)	0 (–)
Other (*n* = 4)	0.03 (0.02)
**Proportion of appropriate MRC of all maternal comments made, 24 months**	
White (*n* = 93)	0.03 (0.03)
Black (*n* = 18)	0.03 (0.02)
Latina (*n* = 3)	0.03 (0.01)
Other (*n* = 5)	0.06 (0.07)
**Proportion of appropriate MRC of all maternal comments made, 36 months**	
White (*n* = 81)	0.04 (0.02)
Black (*n* = 14)	0.03 (0.02)
Latina (*n* = 1)	0.07 (–)
Other (*n* = 1)	0.01 (–)

*There were no significant differences between mothers in their use of appropriate mind-mindedness. Sample sizes are small across groups, and descriptive characteristics should be viewed for heuristic value only.*
